# The Bookworld of Medicine and Science

**Published:** 1904-10-01

**Authors:** 


### The Bookworld of Medicine and Science.

Bradshaw's Dictionary of Bathing-Places and Climatic Health-Resorts. By B. Bradshaw. (London and New York: Kegan Paul, Trench, Trübner and Co., Limited. Pp. 372, with maps).

Mr. Bradshaw's new edition will be welcome to all who travel either for health or pleasure. He compresses a large amount of useful information into a small compass, and the arrangement is so simple that the most hopelessly inept person can find this way about in it. The various health-resorts are first placed alphabetically, and the special indications for treatment at the various stations plainly set forth, with the names of the doctors in attendance, the hotels to be inhabited, and, in many cases, an outline of the mode of treatment. In another division of the book diseases are entered, also alphabetically, and on the opposite side of the page the various stations where the ravages of these special diseases may be best combated. Thus we have opposite to rheumatism in all its forms a list of no fewer than 72 places. In so useful a book it is difficult to discover faults, but it would seem desirable that more hotels should be mentioned. It is annoying to look up such a place as Salsomaggiore, and find no hotels given at all, and only the bald information that "there are many well-appointed hotels and boarding-establishments;" timid travellers like to know beforehand where they are going and what they will have to pay.

